# Supplementary material for: WordCommentsAnalyzer: A windows software tool for qualitative research
Source: F1000Res. 2018 Sep 4;7:536. Originally published 2018 May 3. [Version 2] doi: 10.12688/f1000research.14819.2 (PMC6137411; doi:10.12688/f1000research.14819.2)
Supplement: Supplementary file 3 [file f1000research-7-17614-s0002.tgz › b2a79496-4ada-47c4-9682-a99ce867836d.zip!/Eight Iranian Health Journals/Archives of Rehabilitation_44.docx]

© Archives of Rehabilitation. This work is licensed under CC BY-NC 4.0 (https://creativecommons.org/licenses/by-nc/4.0/).

Volume & Issue: دوره 9 - شماره 3 و 4

Title: مقایسه اثر سه نوع ارتز بر درد پلانتارفاشیاتیس

http://rehabilitationj.uswr.ac.ir/article-1-285-fa.html

هدف: پلانتارفاشیاتیس از شایعترین عوارض پا می‌باشد که به دلیل التهاب فاسیای پلانتار پا در محل اتصال به پاشنه بروز می‌کند. این مطالعه با هدف مقایسه تأثیر سه ارتز پد پاشنه سیلیکونی، ارتز ترموپلاستیک حمایت‌کننده قوس ‌طولی و اسپلینت شبانه‌ کشش‌دهنده نیام‌ کف‌پایی و تاندون آشیل، بر درد پلانتار فاشیاتیس انجام شد. روش بررسی: در این مطالعه نیمه‌تجربی با استفاده از نمونه‌گیری ساده و از جامعه در دسترس، تعداد 16 بیمارمبتلا به پلانتارفاشیاتیس که از بیمارستان شریعتی تهران به مرکز ارتوپدی‌فنی صبا ارجاع شده بودند، انتخاب شدند. محدودیتی از نظر سن، ‌جنس، شاخص توده بدنی،‌ شغل و میزان سطح فعالیت افراد برای ورود به مطالعه وجود نداشت. افراد دارای بیماری‌های سیستمیک و سابقه انجام جراحی در پا از مطالعه حذف شدند. بیماران به ترتیب ورود به مطالعه در یکی از سه گروه درمانی قرار گرفتند. شدت درد بیماران، علاوه ‌بر مراجعه اول، در هفته‌های 2 و 6 و 12 بعد از مراجعه اول، طی سه ماه با استفاده از مقیاس‌های رتبه‌ای- نمره‌ای و رتبه‌ای- کلامی به صورت تلفنی، پیگیری و ثبت ‌شد. در این مطالعه از تحلیل واریانس یک‌طرفه، ضریب همبستگی پیرسون و اسپیرمن، آزمون کروسکال والیس و آزمون اندازه‌گیری مکرر استفاده شد.  یافته‌ها: ضریب همبستگی بین نمره شدت درد عددی و رتبه‌ای بالای 0/80 بود. میانگین شدت درد صبحگاهی (P=0/87)، شامگاهی (P=0/198) و بدترین شدت درد (P=0/113) قبل از ورود به مطالعه در هر سه گروه یکسان بود. هر سه ارتز مذکور بر کاهش درد صبحگاهی، شامگاهی و بدترین شدت درد در طی روز تأثیر داشتند (P<0/001)، اما اختلاف معناداری بین سه گروه درمانی در میزان تأثیر ارتزها و کاهش درد صبحگاهی (P=0/483)، شامگاهی (P=0/462) و بدترین شدت درد (P=0/498) وجود نداشت. نتیجه‌گیری: کاربرد هر یک از سه ارتز یاد شده، به تنهایی و بدون دخالت سایر برنامه های درمانی، بر کاهش درد پلانتارفاشیاتیس تأثیر چشمگیری داشته، ولی هیچ ‌یک از ارتزها نسبت به دو نوع دیگر در کاهش این درد ارجحیت و برتری ندارد.

Title: بررسی رابطه اشارات دستی و گفتار در بزرگسالان دارای لکنت

http://rehabilitationj.uswr.ac.ir/article-1-286-fa.html

هدف: مدت زمان مدیدی است که شاهد همراهی اشارات دست با گفتار هستیم، لیکن راهی که از طریق آن این دو شیوه بیان در تولید با یکدیگر مرتبط می‌شوند، هنوز به طور کامل شناخته نشده است. بنابر این مطالعه حاضر با هدف بررسی ارتباط بین اشاره و گفتار در افراد مبتلا به لکنت و مقایسه آن با افراد سالم انجام گرفته است. روش بررسی: در این مطالعه مقطعی و مقایسه‌ای، 10 فرد بزرگسال مبتلا به لکنت به‌صورت تصادفی از کلینیکهای آسیب شناسی گفتار و زبان انتخاب و با 10 نفر به عنوان گروه کنترل که بر اساس همتاسازی از نظر سن، جنس و میزان تحصیلات با گروه مورد انتخاب شده بودند، مورد مقایسه قرار گرفتند. جهت انجام بررسی مورد نظر از تکلیف بازگویی داستان مربوط به فیلم کارتونی برای برانگیختن اشارات دستی همراه با گفتار استفاده شد. به این ترتیب که از شرکت کنندگان خواسته می‌شد تا به دقت فیلم را مشاهده کرده و داستان آن را با حداکثر جزئیات ممکن در حالی که از آنها ضبط ویدئویی به عمل می‌آمد، برای شنونده‌ای که روبرویشان نشسته تعریف کنند. سپس گفته‌ها و اشارات ضبط شده مورد بررسی و داده‌های حاصل با استفاده از آزمونهای آماری کولموگروف - اسمیرنوف و تی مستقل مورد تجزیه و تحلیل قرار گرفت. یافته‌ها: تحلیل نتایج نشان داد که افراد مبتلا به لکنت نسبت به افراد گروه کنترل از اشارات نمادین کمتری در حین گفتار توصیفی خود استفاده کرده‌اند (P=0/005) همچنین افراد مبتلا به لکنت نسبت به افراد گروه کنترل در بیان هر گفته و به ازای هر واژه نیز از اشارات نمادین کمتری استفاده کرده‌اند (P=0/019). به علاوه بررسی تولید حرکات اشاره حین بروز ناروانی نشان داد که بیش از 70 درصد اشاراتی که همراه با بروز لکنت تولید شده بودند، هم زمان با آن، ثابت و یا گسیخته می‌شدند. نتیجه‌گیری: به نظر می‌رسد اشاره و گفتار، چنان پیوند پیچیده و عمیقی دارند که الگوهای بسامد و زمان بندی مشابهی را نشان داده و کاملاً به موازات یکدیگر پیش می‌روند، به گونه‌ای که نقص در گفتار منجر به نقص در اشارات دستی نیز می‌شود.

Title: بررسی توافق بین دو آزمونگر در استفاده از مقیاس اصلاح‌شده تاردیو برای ارزیابی اسپاستیسیته عضلانی به‌دنبال سکته مغزی

http://rehabilitationj.uswr.ac.ir/article-1-287-fa.html

هدف: اسپاستیسیته یک جزء سندرم نورون محرکه فوقانی است که با افزایش وابسته به سرعت رفلکس‌های کششی تونیک مشخص می‌شود. هدف این تحقیق، ارزیابی پایایی مقیاس اصلاح شده تاردیو از طریق بررسی توافق دو آزمونگر در ارزیابی اسپاستیسیته عضلانی به دنبال سکته مغزی می‌باشد. روش بررسی: در این تحقیق که از نوع تحلیلی و اعتبارسنجی می‌باشد، 30 بیمار دچار فلج اسپاستیک نیمه بدن ناشی از سکته مغزی از بین مراجعین درمانگاه ضایعات اعصاب مرکزی دانشگاه علوم پزشکی تهران مطابق معیارهای لازم به صورت ساده و هدفمند انتخاب شده و دو گروه عضله فلکسور و اکستانسور آرنج آنان با استفاده از یک روش استاندارد و براساس مقیاس اصلاح شده تاردیو ارزیابی شد. ارزیابی‌ها در یک جلسه توسط دو کاردرمانگر انجام شد. ترتیب ارزیابی توسط آزمونگران و ترتیب آزمون عضلات تصادفی بود. پایایی بین دو آزمونگر برای اجزاء مختلف مقیاس که بیانگر سطح اسپاستیسیته می‌باشد، محاسبه شد. آزمونهای ضریب همبستگی درون گروهی (ICC) و کاپا برای ارزیابی پایایی اجزاء مختلف مقیاس استفاده شد.  یافته‌ها: مقادیر ICC برای تفاوت زاویه واکنش عضلانی و زاویه دامنه حرکتی کامل به عنوان معیار اصلی برای تعیین شدت اسپاستیسیته، 0/99 در فلکسورها و 0/95 در اکستانسورها بود. همچنین مقادیر کاپا در بررسی کیفیت واکنش عضلانی در عضلات فلکسور 0/88 و در عضلات اکستانسور 0/89 بود. نتیجه‌گیری: مقیاس اصلاح شده تاردیو در ارزیابی اسپاستیسیته عضلانی پس از سکته مغزی دارای پایایی بالایی در بین دو آزمونگر می‌باشد.

Title: توان‌بخشی به معلولین برای ارتباط با رایانه از طریق تایپ حروف با حرکات چشم

http://rehabilitationj.uswr.ac.ir/article-1-288-fa.html

هدف: امروزه رایانه‌ها عموماً با ابزارهایی نظیر موشواره و صفحه کلید کنترل می‌شوند، در نتیجه افرادی که دچار قطع عضو و یا ناتوانی در ناحیه دستها هستند، از به کارگیری رایانه محروم می‌باشند. در این پژوهش چگونگی طراحی سیستمی که به کمک حرکات چشم، امکان تایپ حروف به افراد معلول را می‌دهد ارائه شده است. روش بررسی: در این طرح مرز بین عنبیه و صلبیه که لیمباس نامیده می‌شود، به کمک فرستنده و گیرنده‌های مادون قرمز ردیابی می‌شود. مقادیر اندازه‌گیری شده از موقعیت چشم به کمک یک نرم‌افزار مورد پردازش قرارگرفته و به مقادیر مناسب جهت حرکت مکان نما برروی حروف قرار داده شده در صفحه نمایشگر، تبدیل می‌شوند. از آنجا که تغییر کاربر و حرکات ناخواسته سر می‌تواند روی عملکرد سیستم تأثیر بگذارد، یک مرحله کالیبراسیون نیز در نظر گرفته شده است تا سیستم بتواند برروی شرایط جدید تنظیم شود. سیستم طراحی شده روی 10 نفر (5 مرد و 5 زن) آزمایش و داده‌‌های حاصل مورد بررسی قرار گرفت. یافته‌ها: با آزمایش سیستم، مشخص گردید که سیستم طراحی شده با دقت حدود 38/3±11/4 سانتی‌متر در تفکیک فواصل، توانایی تشخیص 12 نقطه بر روی صفحه نمایشگر 15 اینچ را دارد. آزمایش‌ها نشان می‌دهد که یک شخص غیر ماهر یک جمله دارای 14 حرف و نماد را در متوسط زمان 3 دقیقه و 30 ثانیه با خطای حدود %28 به کمک حرکات چشمش با این سیستم تایپ می‌کند. این مقادیر برای یک شخص ماهر حدود 3 دقیقه با خطای %13 می‌باشد. به این ترتیب سیستم با قابلیت اطمینان نسبتاً بالایی به کاربر اجازه ارتباط با رایانه را می‌دهد. نتیجه‌گیری: با توجه به قدرت سیستم در تفکیک‌پذیری فواصل، 12 نقطه برروی صفحه نمایشگر برای انجام 12 عملکرد مختلف تعبیه شده است. تعدادی از مشخصه‌های صفحه کلید در صفحه اول و باقی آن‌ها در صفحات بعدی قرار داده شده است. سهولت استفاده، عدم تماس مستقیم سیستم با چشم و بدن کاربر و هزینه بسیار پایین آن نسبت به دیگر سیستم‌های موجود از جمله مزایای سیستم می‌باشند.

Title: مقایسه نیازهای توجهی کنترل پاسچرال ورزشکاران سالم و ورزشکاران مبتلا به بی‌ثباتی عملکردی مچ پا

http://rehabilitationj.uswr.ac.ir/article-1-289-fa.html

هدف: کاهش منابع توجهی در مبتلایان به کاهش یا اختلال اطلاعات حسی دیده شده است. هدف از این مطالعه مقایسه نیازهای توجهی کنترل پاسچرال ورزشکاران مبتلا به بی‌ثباتی عملکردی مچ پا و همتایان سالم می‌باشد. روش بررسی: 15 ورزشکار دانشجوی مبتلا به بی‌ثباتی عملکردی مچ پا و 15 فرد همتای سالم با نمونه‌گیری ساده و در دسترس انتخاب و در این مطالعه مقطعی مورد- شاهدی که بر اساس الگوی تکلیف دوگانه طراحی شد مورد مقایسه قرار گرفتند. ابزار به‌کار رفته سیستم تعادلی بایودکس بود که با استفاده از آن شاخص ثبات کلی، در حالی که آزمودنیها 2 دشواری از تکلیف پاسچرال (شامل سطح ثباتی 5 و 7 از سیستم تعادلی بایودکس) و 2 تحمیل تکلیف شناختی (شامل تحمیل صفر و شمارش معکوس) را تجربه می‌کردند، اندازه گیری شد. به منظور بررسی اثر متقابل متغیرهای مستقل (دشواری تکلیف پاسچرال، دشواری تکلیف شناختی و سطح سلامتی) با متغیر وابسته مورد مطالعه (شاخص ثبات کلی) از آزمون تحلیل واریانس چند عاملی مختلط و جهت مقایسه میانگین متغیر وابسته در دو گروه مورد مطالعه، از آزمون تی مستقل استفاده شد یافته‌ها: آزمون تحلیل واریانـس اثرهای متقـابل سطح سلامتی با دشواری تکلیف پاسچرال و دشواری تکلیف‌شناختی بر شاخص ثباتی کلی را معنی‌دار نشـان داد. به این معنی که بیمـاران در مواجهه با تکـلیف‌شناختی (P=0/01) و ایسـتادن بر سـطح بی‌ثبات‌تر (P=0/03)، افزایش بیشتری در شاخص ثبات کلی به نسبت گروه سالم نشان داده و اختلال پاسچرال بیشتری داشتند. نتیجه‌گیری: به نظر می‌رسد که بی‌ثباتی عملکردی مچ پا با افزایش نیازهای توجهی کنترل نیمه پویای پاسچرال همراه است. بنابراین تحمیل تکلیف شناختی ممکن است بتواند به عنوان یک استراتژی مؤثر در برنامه ورزشی مبتلایان به بی‌ثباتی عملکردی مچ پا منظور گردد.

Title: بررسی تأثیر آب‌درمانی بر کیفیت زندگی بیماران زن مبتلا به مولتیپل اسکلروزیس

http://rehabilitationj.uswr.ac.ir/article-1-290-fa.html

هدف: مولتیپل اسکلروزیس یکی از شایعترین بیماریهای مزمن سیستم عصبی مرکزی و از مهمترین بیماریهای تغییردهنده زندگی فرد، به ویژه در سنین جوانی می‌باشد که باعث افت شدید سطح کیفیت زندگی بیماران مبتلا شده و به تدریج فرد را به سمت ناتوانی پیش خواهد برد. این پژوهش با هدف بررسی تأثیر آب‌درمانی به عنوان یک روش درمانی مکمل، بر کیفیت زندگی بیماران مبتلا به مولتیپل اسکلروزیس انجام شده است. روش بررسی: در این مطالعه شبه تجربی،50 نفر از زنان مبتلا به مولتیپل اسکلروزیس از انجمن ام‌.اس. به صورت ساده و دردسترس انتخاب و به‌ صورت تصادفی تعادلی به دو گروه شاهد و آزمون تخصیص داده شدند. پس از یک جلسه آشناسازی بیماران گروه آزمون با اهداف و روش مداخله، 24 جلسه آب‌درمانی برای آنها در مدت سه ماه انجام شد، در صورتی که برای گروه شاهد هیچگونه مداخله‌ای انجام نشد. ابزارهای جمع آوری اطلاعات شامل پرسشنامه اطلاعات دموگرافیک، فرم کوتاه کیفیت زندگی (8SF-) و چک‌لیست‌های خود گزارش دهی بود. از آزمون‌های آماری کای دو، تی زوجی، تی مستقل، تحلیل واریانس با اندازه‌گیریهای تکراری و ضریب همبستگی جهت تجزیه و تحلیل داده‌ها استفاده گردید. یافته‌ها: بین دو گروه در میانگین امتیاز کل (P=0/81) و امتیازات ابعاد جسمی (P=0/4) و روانی (P=0/21) کیفیت زندگی قبل از مداخله تفاوت معناداری وجود نداشت، ولی بعد از مداخله میانگین امتیاز کل (P<0/0001) و امتیازات ابعاد جسمی (P<0/001) و روانی (P=0/003) کیفیت زندگی بین دو گروه شاهد و آزمون تفاوت معنا‌دار داشت. همچنین آزمون آماری آنالیز واریانس با اندازه‌گیری‌های مکرر تفاوت معنا‌داری را در میانگین امتیاز کل (P=0/04) و امتیازات ابعاد جسمی (P=0/05) و روانی (P=0/04) کیفیت زندگی در چهار بار اندازه گیری بین دو گروه نشان داد. نتیجه‌گیری: انجام روش آب‌درمانی با عث ارتقاء سطح کیفیت زندگی بیماران مولتیپل اسکلروزیس می‌گردد، لذا انجام این تکنیک به عنوان یک روش مؤثر و مقرون به صرفه در هزینه و زمان و همچنین داشتن قابلیت یادگیری و اجرای آسان به بیماران مولتیپل اسکلروزیس توصیه می‌گردد.

Title: مقایسه تأثیر آموزش خواندن و آموزش سنتی زبان‌درمانی بر زبان درکی و بیانی کودکان مبتلا به نشانگان داون

http://rehabilitationj.uswr.ac.ir/article-1-291-fa.html

هدف: با توجه به اینکه کودکان مبتلا به سندرم داون دارای تأخیر شدید رشد زبانی بوده و به نظر نمی‌رسد که زبان درمانی با تکیه بر مسیر شنوایی ـ واجی تأثیر مطلوبی در رشد مهارت‌های زبانی این کودکان ایجاد کند، لذا پژوهش حاضر به مقایسه تأثیر آموزش خواندن و آموزش سنتی بر زبان درکی و بیانی کودکان مبتلا به سندرم داون 4 تا 10 ساله با بهره هوشی 40 تا 60 می‌پردازد. روش بررسی: در این پژوهش شبه تجربی و مداخله‌ای با استفاده از نمونه‌گیری در دسترس و ساده و بر اساس معیارهای لحاظ‌شده، از کلینیک‌های توانبخشی نوید عصر تهران، رضوان شهریار، احسان کرج و انجمن سندرم داون کرج 20 کودک مبتلا به سندرم داون از میان 96 کودک سندرم داون انتخاب و به صورت تصادفی تعادلی به دو گروه 10 نفره تخصیص یافتند. گروه اول تحت آموزش خواندن از نوع کل خوانی و گروه دوم تحت آموزش سنتی به مدت شش ماه، سه جلسه در هفته و هر جلسه پانزده دقیقه قرار گرفتند. شرایط زبانی هر دو گروه به صورت پیش‌آزمون و پس‌آزمون در زمینه بهره‌های زبانی درکی با استفاده از آزمون رشد زبان فارسی سنجیده شد. داده‌های حاصل با استفاده از آزمون‌های آماری کولموگروف - اسمیرنوف، تی ـ مستقل و تی ـ زوجی مورد تحلیل قرار گرفت. یافته‌ها: هر دو گروه پیش از آموزش در تمام بهره‌های زبانی اعم از زبان درکی (P=0/452)، بیانی (P=0/1) و گفتاری (P=0/428) شرایط یکسانی داشتند، اما پس از آموزش شرایط زبانی گروه خواندن در هر سه متغیر فوق، یعنی زبان درکی (P=0/043)، بیانی (P<0/001) و گفتاری (P<0/001) نسبت به گروه دیگر به میزان معنی‌داری رشد و پیشرفت بیشتری نشان داد. نتیجه‌گیری: آموزش خواندن به روش کل خوانی نسبت به آموزش سنتی تأثیر بهتر و بیشتری بر بهره زبان درکی، بیانی و گفتاری کودکان مبتلا به نشانگان داون می‌گذارد. به عبارتی بررسی فنوتیپ رفتاری و شناختی کودکان داون نشان می‌دهد که مسیر بینایی بیشتر از مسیر شنیداری، یادگیری زبان گفتاری را تسهیل می‌کند و می‌توان با رویکرد آموزش استعدادها بهترین نتایج را بدست آورد.

Title: رابطه همبودی اختلالات شخصیت کلاستر B و اختلال افسردگی اساسی با عود افسردگی

http://rehabilitationj.uswr.ac.ir/article-1-292-fa.html

هدف: هدف از این مطالعه تعیین رابطه همبودی اختلالات شخصیت کلاستر B و افسردگی اساسی با عود افسردگی می‌باشد. روش بررسی: در این مطالعه تحلیلی و مقایسه‌ای 100 بیمار مبتلا به افسردگی اساسی که در طی 5 سال گذشته افسردگی اساسی را تجربه کرده و در زمان پژوهش در دوره بهبودی نسبی به سر می‌بردند، به روش نمونه‌گیری هدفمند از مراجعان به چند مرکز سرپایی و درمانگاهی تهران انتخاب شدند. افرادی که بیش از دو بار تجربه عود داشته در گروه مورد و بیماران بدون تجربه عود در گروه گواه قرار گرفته و دو گروه از نظر متغیر‌های دموگرافیک همتاسازی شدند. جمع‌آوری داده‌ها با استفاده از مصاحبه ساختار یافته تشخیصی به منظور ارزیابی اختلالات شخصیت کلاستر B، پرسشنامه افسردگی بک و پرسشنامه مشخصات دموگرافیک انجام و با آزمونهای آماری مجذور خی، ضریب همبستگی اسپیرمن و تی مستقل مورد تحلیل قرار گرفت. یافته‌ها: همبودی اختلال شخصیت مرزی به میزان معناداری در افراد افسرده دارای سابقه عود بیشتر است (P<0/001). همبودی اختلال خودشیفته نیز بین دو گروه تفاوت معناداری داشته (P=0/016)، ولی همبودی اختلال شخصیت نمایشی بین دو گروه تفاوت معناداری نداشت (P=0/401). نتیجه‌گیری: بر اساس تفاوت معنادار همبودی اختلالات شخصیت مرزی و خودشیفته در دو گروه و نقشی که این اختلالات در عود افسردگی ایفا می‌کنند، در آینده طرح‌ریزی یک درمان مؤثر برای افسردگی مستلزم در نظر گرفتن مؤلفه‌های شخصیت در کنار نشانگان افسردگی می‌باشد.

Title: میزان آگاهی دانش‌آموزان دبیرستانی منطقه 9 تهران از اثرات قرص‌های جنون‌آور (اکس)

http://rehabilitationj.uswr.ac.ir/article-1-293-fa.html

هدف: در میان مواد مخدر، ظهور قرص‌های روانگردان (اکستاسی) خطر جدیدی است که طرفداران ویژه‌ای میان نوجوانان و جوانان دارد. به نظر می‌رسد با توجه به جدید بودن قرص‌های اکستاسی و نامتشابه بودن عوارض مصرف آن با سایر مواد مخدر، میزان آگاهی و شناخت افراد جامعه مورد مطالعه از آن کم باشد، لذا هدف این تحقیق شناخت میزان آگاهی دانش‌آموزان از اثرات قرص‌های جنون‌آور است. روش بررسی: این پژوهش توصیفی و مقطعی بر روی 154 دانش‌آموز پایه سوم دبیرستان از منطقه 9 تهران که به روش نمونه‌گیری تصادفی ساده انتخاب شده و میزان آگاهی آنان در زمینه موضوع تحقیق با استفاده از پرسشنامه محقق ساخته سنجیده شده صورت گرفت. روایی پرسشنامه از طریق روایی صوری با نظر متخصصین و پایایی آن با استفاده از ضریب آلفای کرونباخ و ارزیابی همسانی درونی سئوالات انجام و مقدار ضریب 0/85 بود که مطلوب می‌باشد. برای تحلیل داده‌ها از ضریب همبستگی و آزمون مان ویتنی استفاده شد. یافته‌ها: دانش‌آموزان به 14/7 درصد پرسش‌ها پاسخ غلط و به 38 درصد آنها پاسخ نمی‌دانم داده و هر دانش‌آموز به طور متوسط به 2 سئوال پاسخ غلط داده و پاسخ درست را به‌خوبی نمی‌دانست. میزان آگاهی کلی دانش‌آموزان دختر و پسر اختلاف معنادار نداشت (P=0/573). همچنین رابطه معناداری بین میزان آگاهی دانش‌آموزان از آثار مصرف و عوارض سوء قرص اکس و جنسیت آنان وجود ندارد (P=0/572). نتیجه‌گیری: میزان آگاهی و شناخت دانش‌آموزان از علائم مصرف و عوارض سوء قرص‌های اکس در سطح پایینی (47/3 درصد) قرار دارد.

Title: مقایسه رضایت مراجعین از مراکز توانبخشی سرپایی دولتی و خصوصی استان کردستان

http://rehabilitationj.uswr.ac.ir/article-1-294-fa.html

هدف: در این پژوهش میزان رضایت مراجعین به مراکز توانبخشی دولتی و خصوصی استان کردستان از این مراکز بررسی و با هم مورد مقایسه قرار گرفت. روش بررسی: در این مطالعه مقطعی – مقایسه‌ای، 415 نفر از مراجعین بار اول مراکز دولتی و خصوصی سرپایی توانبخشی استان کردستان طی شش ماهه دوم سال 1383 به شیوه نمونه گیری تصادفی ساده و طبقه بندی شده انتخاب گردیدند. اطلاعات بر اساس پرسشنامه ساختار یافته طی مصاحبه تلفنی جمع آوری و رضایت هر فرد بر اساس نمره‌ای بین صفر تا بیست مورد سنجش قرار گرفت. اعتبار صوری پرسشنامه توسط اساتید فن مورد تأیید قرار گرفته و ضریب پایائی آن براساس آزمون - بازآزمون 0/83=r و ضریب همبستگی داخلی آن بر اساس آلفای کرونباخ 0/76 برآورد شد. تجزیه و تحلیل داده‌های حاصل با استفاده از آزمونهای آماری مجذور خی و تی مستقل انجام شد.  یافته‌ها: میانگین نمره رضایت کلی مراجعین مراکز دولتی (1/6±12/48) از خصوصی (1/4±12/15) بیشتر و تفاوت آنها معنی‌دار بود (P=0/04). مشتریان مراکز خصوصی از وضعیت فیزیکی (P<0/001)، دسترسی زمانی (P=0/018) و نحوه برخورد کارکنان مراکز (P=0/008)، رضایتی بیش از مشتریان مراکز دولتی داشتند که البته در هیچ مورد تفاوت بیش از یک نمره نبود. در مقابل مشتریان مراکز دولتی از دسترسی فیزیکی (P=0/01)، دسترسی مالی (P<0/001) و کیفیت خدمات (P<0/001) رضایت بیشتری ابراز کردند. نتیجه‌گیری: با وجودی که تفاوت بین میزان رضایت مراجعین مراکز دولتی و خصوصی از نظر آماری معنادار است، اما بجز در مورد دسترسی مالی چشمگیر نیست. ارتقاء کیفیت خدمات توانبخشی و کسب رضایت بیشتر مراجعین هم برای مراکز دولتی و هم برای مراکز خصوصی امری ضروری است. گسترش پوشش بیمه‌ای به بخش خصوصی پیشنهاد می‌شود.

Title: موانع مشارکت بیماران سکته قلبی در برنامه بازتوانی قلبی

http://rehabilitationj.uswr.ac.ir/article-1-295-fa.html

هدف: بازتوانی قلبی باعث افزایش کیفیت زندگی، کاهش سکته مجدد قلبی، بهبود عملکرد جسمی و وضعیت روانی بیماران می‌گردد. مطالعه حاضر با هدف شناسایی موانع مشارکت بیماران سکته قلبی در برنامه بازتوانی قلبی صورت گرفت. روش بررسی: این مقاله گزارش بخشی از مطالعه‌ای است که به روش تئوری پایه انجام شد. هیجده نفر بیمار مبتلا به سکته قلبی، 3 نفر پزشک متخصص قلب، و 3 نفر پرستار بخش سی سی یو، به صورت نمونه گیری هدفمند و نظری انتخاب شدند. مصاحبه نیمه ساختار یافته روش اصلی جمع آوری داده‌ها بود و تا زمان اشباع داده‌ها ادامه یافت. داده‌ها به روش تئوری پایه اشتراوس و کوربین (1998) تحلیل شد. اعتبار داده‌ها بر اساس معیارهای پیشنهادی لینکولن و گوبا تأیید گردید. یافته‌ها: اکثر بیماران در برنامه‌های بازتوانی قلبی شرکت نکرده‌اند. موانع مشارکت بیماران در برنامه‌های بازتوانی قلبی درچهار طبقه عوامل فردی، اجتماعی- فرهنگی، اقتصادی و عوامل مربوط به نظام درمان و سلامت طبقه‌بندی گردید. نتیجه‌گیری: بسیاری از موانع مشارکت بیماران در برنامه‌های بازتوانی قابل تعدیل هستند و تغییر آنها زمینه حضور بیماران را در برنامه‌های بازتوانی قلبی تسهیل نماید.
